# Preliminary biological evaluation of ^18^F-AlF-NOTA-MAL-Cys-Annexin V as a novel apoptosis imaging agent

**DOI:** 10.18632/oncotarget.16994

**Published:** 2017-04-10

**Authors:** Chunxiong Lu, Quanfu Jiang, Minjin Hu, Cheng Tan, Huixin Yu, Zichun Hua

**Affiliations:** ^1^ Key Laboratory of Nuclear Medicine, Ministry of Health & Jiangsu Key Laboratory of Molecular Nuclear Medicine, Jiangsu Institute of Nuclear Medicine, Wuxi 214063, China; ^2^ Jiangsu Target Pharma Laboratories Inc., Changzhou High-Tech Research Institute of Nanjing University, Changzhou 213164, China; ^3^ The State Key Laboratory of Pharmaceutical Biotechnology, Nanjing University, Nanjing 210093, China

**Keywords:** site-specific labeling, ^18^F-AlF-NOTA-MAL-Cys-Annexin V, apoptosis imaging

## Abstract

A novel annexin V derivative (Cys-Annexin V) with a single cysteine residue at its C-terminal has been successfully labeled site-specifically with NOTA-maleimide aluminum [^18^F]fluoride complexation and evaluated it as a novel apoptosis agent *in vitro* and *in vivo*. The total synthesis time of ^18^F-AlF-NOTA-MAL-Cys-Annexin V from [^18^F]fluoride was about 65 min. The tracer was stable *in vitro* and it was excreted through renal in normal mice. The rate of the tracer bound to erythrocytes with exposed phosphatidylserine was 89.36±0.61% and this binding could be blocked by unlabeled Cys-Annexin V. In rats treated with cycloheximide, there were 6.23±0.23 times (n=4) increase in hepatic uptake of the tracer as compared to normal rats at 1h p.i. The uptake of the tracer in liver also could be blocked by co-injection of unlabeled Cys-Annexin V. These results indicated the favorable characterizations such as convenient synthesis and specific apoptotic cells targeting of^18^F-AlF-NOTA-MAL- Cys-Annexin V were suitable for its further investigation in clinical apoptosis imaging.

## INTRODUCTION

Apoptosis, also known as programmed cell death, is an important way to maintain the relative balance of the body, which is also closely related with a variety of pathological processes such as myocardial ischemia and tumor response to treatment [1, 2]. Therefore, it is very important to detect and quantify the apoptosis *in vivo* in order to diagnose and evaluate therapeutic efficacy.

During the early phase of apoptosis, phosphati-dylserine (PS) in the lipid bilayer of the cell membrane is flipped from the inner layer to the outer layer and exposed to the cell surface [3, 4]. So it is a good target for the development of probe to image apoptotic cells [5]. Annexin V with high-affinity for PS is an endogenous protein with a molecular weight of about 36-kD and contains about 319 amino acids. It belongs to the calcium-dependent phospholipid-binding protein family and always used as PS targeting agent [6, 7]. Flow cytometry or fluorescence microscopy examination of apoptosis using fluorescein or biotin-labeled Annexin V as a probe is a sensitive, efficient, mature laboratory testing method. Annexin V labeled with various radionuclides are also useful as radiotracers *in vivo* imaging of apoptosis as single photon emission computed tomography (SPECT) and positron emission tomography (PET) imaging agents. Annexin V and Annexin V derivatives were radiolabeled with ^125^I, ^123^I, ^111^In and ^99m^Tc for SPECT imaging of apoptosis [8–11]. In 1998, ^99m^Tc-HYNIC-Annexin V was first reported by Blankenberg et al and then it became the most successful and extensively studied radiotracer of SPECT apoptosis imaging. [12]. In a wide array of preclinical [11, 13] and clinical studies [14–16]^99m^Tc-HYNIC-Annexin V imaging was useful to assess tumor response to therapy.

Since PET is more sensitive and quantitative than SPECT, Annexin V has been radiolabeled with positron emission radioisotopes including ^124^I [17–18], ^68^Ga [19], and ^18^F [20–22] for PET imaging. Among them ^18^F-labeled Annexin V was the most studied radiotracer because of the favorable properties of ^18^F, such as moderate half-life of 109.8 min, high image resolution and lower radiation dose [23].

NH_2_ group reactive agent N-succinimidy- 4-^18^ F-fluorobenzoate (^18^F-SFB) was used mostly as prosthetic group to label Annexin V [20, 22, 24], however the reaction between ^18^F-SFB and Annexin V is nonspecific. ^18^F-SFB could react with any NH_2_ group of Annexin V, whereas there are 23 NH_2_ groups available on Annexin V. N-substituted maleimides were used mainly as thiol reactive agents to radiolabel proteins at free thiol of cysteines [25]. We previously evaluated one fluorine-18-labeled analog of Annexin V mutant, Cys-Annexin V, having a C-terminal cysteine, prepared by radiolabeling with ^18^F-FBEM [26]. ^18^F-FBEM-Cys-Annexin V displayed good liver uptake in rats treated with cycloheximide, however the preparation process of the radiotracer consumed much time. To overcome this difficulty, we developed a new method to radiolabel Cys-Annexin V using aluminum [^18^F]fluoride (^18^F-AlF) with a maleimide monoamide NOTA (NOTA-MAL). This method was required less time than synthesis of ^18^F-FBEM-Cys-Annexin V. It is important to reduce synthesis time of radiotracer, because of the physical half-life of ^18^F is only 109.8 min and also it is helpful to reduce radiation exposure.

The aim of this study was to evaluate ^18^ F-AlF-NOTA-MAL-Cys-Annexin V as a new apoptotic imaging agent *in vitro* and *in vivo*.

## RESULTS

### Chemistry and radiochemistry

**Figure 1:**
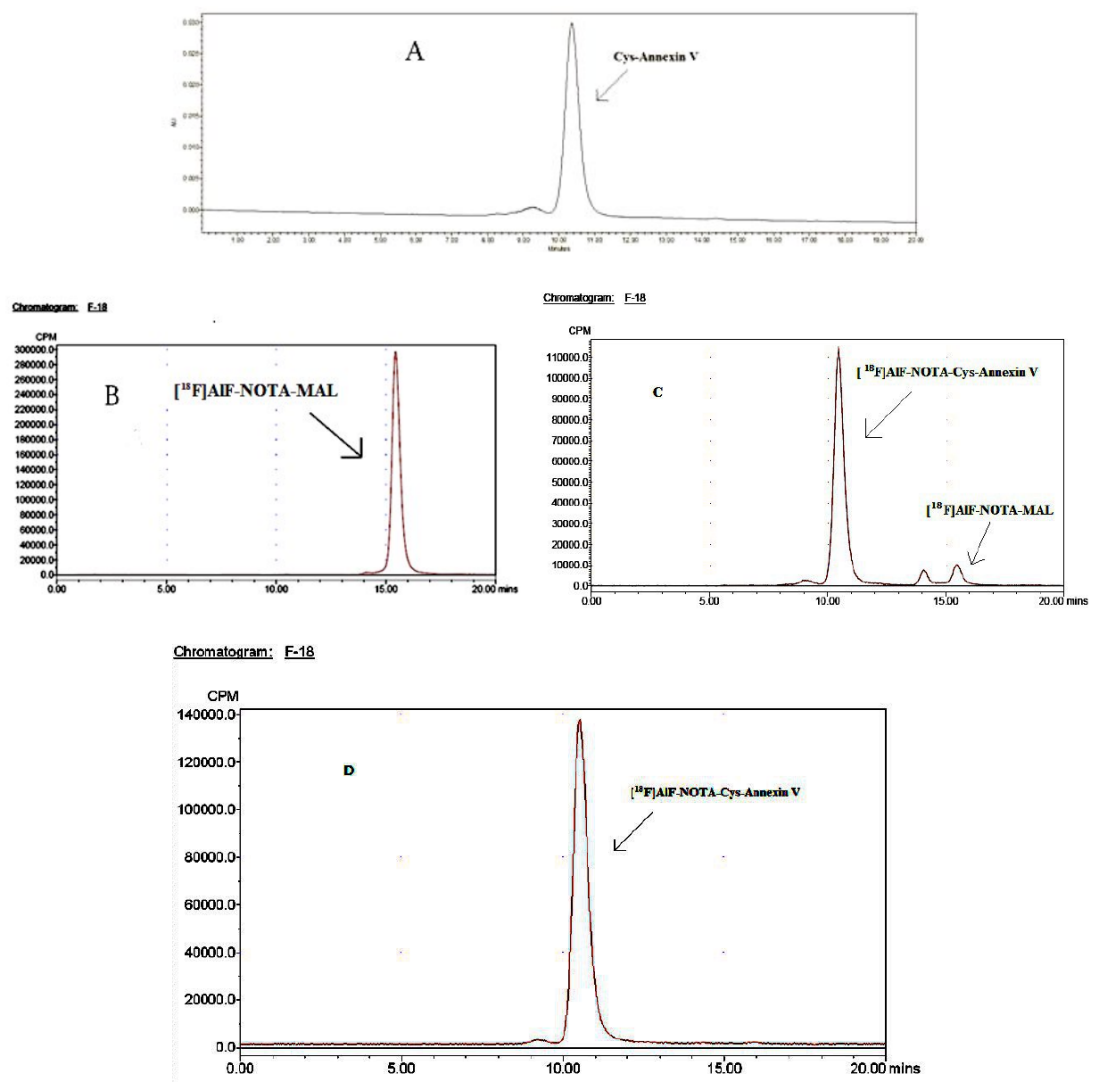
^18^F-AlF-NOTA-MAL-Cys-Annexin V and ^18^F-AlF-NOTA-MAL could be separated completely HPLC chromatogram (isocratic, 0.05 mol/L phosphate buffer (pH=7.0), flow 1.0 mL/min) of: **(A)** Cys-Annexin V, t_R_=10.5 min (UV), HPLC radiochromatograms of **(B)** ^18^F-AlF-NOTA-MAL, t_R_=15.7 min, **(C)** reaction mixture (^18^F-AlF-NOTA-MAL-Cys-Annexin V, t_R_=10.6 min, ^18^F-AlF-NOTA-MAL, t_R_=15.7 min) and **(D)** ^18^F-AlF-NOTA-MAL-Cys-Annexin V, t_R_=10.6 min.

In Figure 1, the retention times of Cys-Annexin V, ^18^F-AlF-NOTA-MAL-Cys-Annexin V and ^18^F-AlF-NOTA-MAL were 10.5 min 10.6 min and 15.7 min, respectively. This means that ^18^F-AlF-NOTA-MAL- Cys-Annexin V and Cys-Annexin V were consistent, and ^18^F-AlF-NOTA-MAL- Cys-Annexin V could be completely separated with ^18^F-AlF-NOTA-MAL. The total synthesis time of ^18^F-FBEM-Cys-Annexin V from [^18^F]fluoride was over 120 min [26], however that of ^18^F-AlF-NOTA-MAL-Cys- Annexin V was just about 65 min. According to HPLC analysis, the radiochemical purity and radiolabeling yield of ^18^F-AlF-NOTA-MAL-Cys- Annexin V were 97.38±0.35% and 78.88±5.23% (based on the starting ^18^F-AlF-NOTA-MAL, non-decay corrected, n=5), respectively. The specific activity of ^18^F-AlF-NOTA-MAL and ^18^F-AlF-NOTA-MAL-Cys-Annexin V were above 3.17GBq/µmol and 54.0GBq/µmol, respectively.

### *In vitro* stability of ^18^F-AlF-NOTA-MAL-Cys-Annexin V

**Figure 2:**
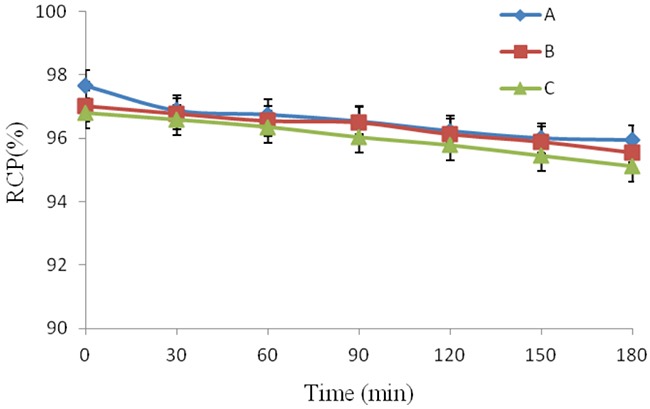
*In vitro* ^18^F-AlF-NOTA-MAL-Cys-Annexin V was stable at different intervals in **(A)** PBS, **(B)** human serum and **(C)** cell culture media.

It is important to study the stability of radiotracer. If radiotracer is not stable, some radioactive decomposed side products will affect the imaging results. The stability of ^18^F-AlF-NOTA-MAL-Cys-Annexin V was studied in (A) phosphate buffered saline, (B) human serum and (C) cell culture media, respectively. The results are presented in Figure 2. ^18^F-AlF-NOTA-MAL-Cys-Annexin V was stable in PBS, human serum and cell culture media and the radiochemical purities of the tracer were also >95% with HPLC analysis after 180 min. These results suggested that the radiotracer was stable *in vitro*.

### Bioactivity study

**Figure 3:**
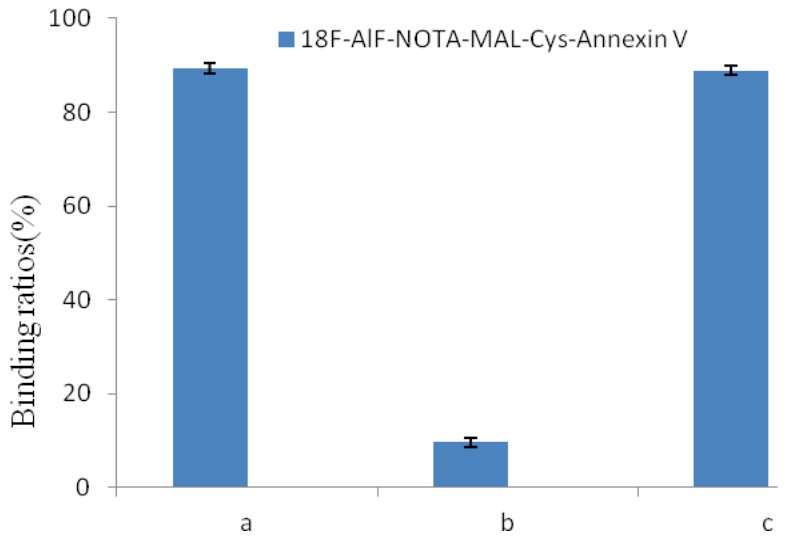
Radioactivity bound to erythrocytes (%) of ^18^F-AlF-NOTA-MAL-Cys-Annexin V in the presence of **(A)** 0-fold unlabeled Cys-Annexin V, **(B)** 50-fold unlabeled Cys-Annexin V and **(C)** 50-fold BSA.

To determine the bioactivity of the radiotracer in a cell binding assay, the rate of it bound to erythrocytes was 89.36±0.61% as shown in Figure 3. When tubes were added with 50-fold of Cys-Annexin V, the rate of it bound to erythrocytes was 9.58 ± 1.06%. On the other hand, when the addition with 50-fold of BSA, binding of ^18^F-AlF-NOTA-MAL-Cys-Annexin V was 88.84 ± 1.01 %. These values indicated that ^18^F-AlF-NOTA-MAL-Cys-Annexin V was specific binding to PS of erythrocytes.

### Dynamic MicroPET imaging of mice

**Figure 4:**
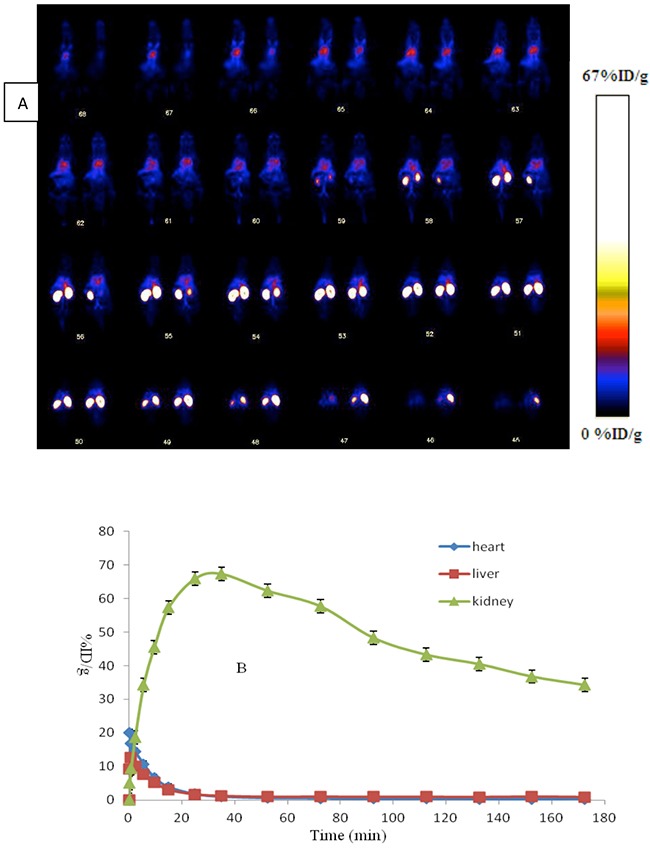
**(A)** Whole body coronal microPET images of ICR mouse from a 0-180 min dynamic scan after injection of 3.7 MBq ^18^F-AlF-NOTA-MAL-Cys-Annexin V. **(B)** Quantified time-activity curves of major organs (liver, heart and kidney) after injection of 3.7 MBq ^18^F-AlF-NOTA-MAL-Cys-Annexin V in normal ICR mice (*n* = 4).

After administration of ^18^F-AlF-NOTA-MAL-Cys-Annexin V, major organ time-activity curves were obtained from 0 to 180 min dynamic microPET scans. In Figure 4, diamond represents heart, triangle represents kidney and square represents liver. The radioactivity kinetics were calculated from a region-of-interest analysis of the dynamic microPET scans. The radiotracer was excreted mostly through the kidney and the peak (67% ID/g) at about 35 min p.i. and then reduced to 34% ID/g at 180 min p.i.

### Apoptotic rat liver imaging

**Figure 5:**
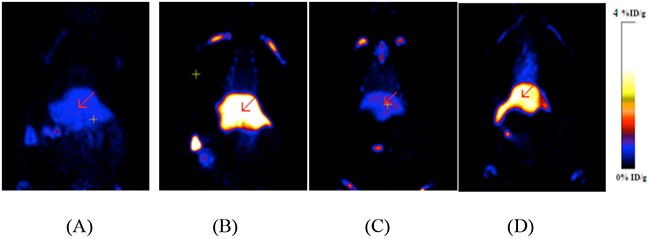
MicroPET images of 18F-AlF-NOTA--MAL-Cys- Annexin V at 1 h p.i. **(A)** Control group; **(B)** Treated group; **(C)** Blocking group; **(D)** BSA group. High radiouptake in liver (arrow) of B and D.

Twelve liver apoptosis rats were induced with 10 mg/kg cycloheximide and divided into three groups, treated group(B), blocking group(C) and BSA group(D). The other four normal rats were served as control group(A). In Figure 5. four representative coronal microPET images displayed of 7.4 MBq ^18^F-AlF-NOTA-MAL-Cys-Annexin V at 1h p.i. The uptakes of the radiotracer in liver (arrow) of control group, treated group, blocking group and BSA group were 0.50±0.02, 3.09±0.08, 0.76±0.04 and 3.34±0.09%ID/g, respectively, at 1h p.i. The uptake ratios (treated/control, blocking/control, BSA/control) of liver were 6.23±0.23, 1.52±0.07, 6.72±0.21(n=4), respectively, at 1h p.i. These values means that the uptake of ^18^F-AlF-NOTA-MAL-Cys-Annexin V was increased in the cycloheximide treatment liver and it also could be blocked with unlabeled Cys-Annexin V and BSA could not block the tracer uptake in liver of rats. These results indicated ^18^F-AlF-NOTA--MAL-Cys-Annexin V could bind specifically to apoptotic cells.

**Figure 6:**
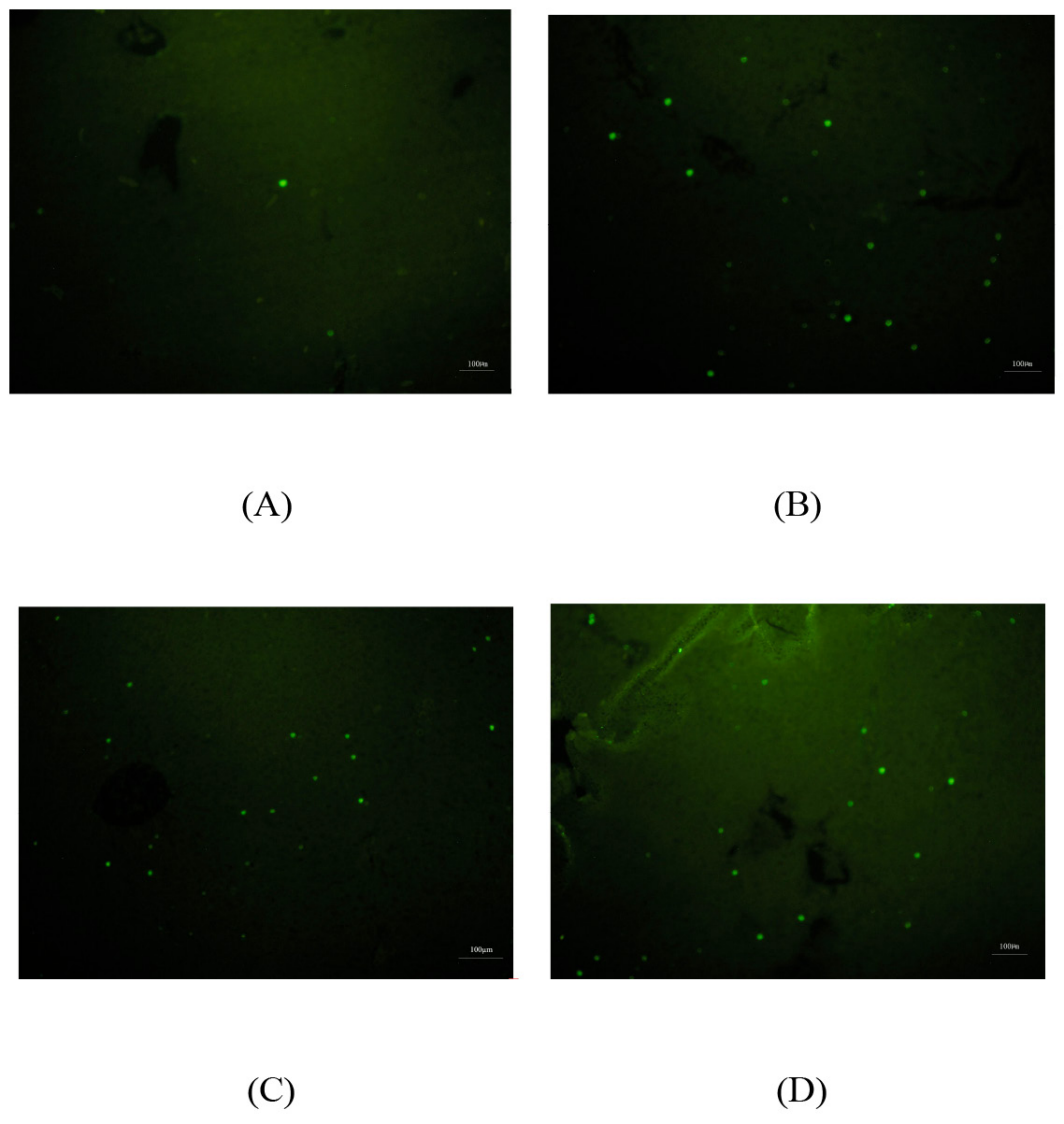
TUNEL staining of liver specimen in Control group **(A)**; Treated group **(B)**; Blocking group **(C)** and BSA group **(D)**. Green dot represents positive TUNEL staining. Scale bar=100µm.

In Figure 6, four representative images of liver TUNEL staining sections were displayed. There were little apoptotic nuclei (green nuclei) in rats’ liver of control group and there were more apoptotic nuclei in that of treated group (B), blocking group (C) and BSA group (D). The rates of TUNEL-positive nuclei were 2.0±0.3%, 15.2±1.5%, 14.8±0.5% and 15.5±1.2% of control group, treated group, blocking group and BSA group, respectively. The liver uptake ratio as measured via microPET at 1 h p.i. between treated group and control group correlated well with the ratio of apoptotic nuclei in liver measured by using TUNEL staining between treated group and control group.

## DISCUSSION

Site-specific labeling method is important for providing a chemically homogeneous radioactive conjugate with defined *in vivo* properties. Since there is no free thiol group provided by cysteine in the Annexin V molecule, we introduce a unique cysteine residue at the desired position and use thiol-mediated chemical labeling or chelator coupling. The Annexin V derivative with a single cysteine residue at its C-terminal (Cys-Annexin V) has been successfully labeled with ^18^F-FBEM by this approach [26].^18^F-FBEM-Cys-Annexin V mainly excreted through the renal pathway in nornal mice and showed high uptake in the rats’ liver treated with cycloheximide. Despite the encouraging results for ^18^F-FBEM-Cys-Annexin V, the radiosynthesis of [^18^F]FBEM was time consuming, which has became the impediment of this radiotracer to widespread use.

Compared with DOTA, NOTA is a promising chelator to provide more stable complexes with a number of radiometals such as gallium and indium [27, 28]. A refined method has been reported by using a complex composed of NOTA and [^18^F]aluminum fluoride to label peptides within a short time about 10min [29–31]. These results suggested that NOTA is a very promising chelator for radiolabeling of proteins and peptides.

Our procedure for preparation of ^18^F-FBEM-Cys-Annexin V required [^18^F]FBEM, which was synthesized by an semiautomated synthesis device and then purified by HPLC [26]. The synthesis process of ^18^F-FBEM was complex. First, the precursor in anhydrous acetonitrile was reacted with [^18^F]fluoride under conditions of dried Kryptofix2.2.2 and K_2_CO_3_ at 100°C for about 10 min. The intermediate of ^18^F-fluorobenzoic acid (^18^F-FBA) was obtained by hydrolysis with NaOH, acidification with HCl and purification with C18 Sep-Pak column. Second, The mixture of ^18^F-FBA, N-(2-aminoethyl) maleimide, diethyl cyanophosphonate, and N,N-diisopropylethyl amine in anhydrous acetonitrile was heated to obtain crude ^18^F-FBEM. Third, the purification of ^18^F-FBEM was performed by semi-preparative HPLC. Compared to ^18^F-FBEM, synthesis of ^18^F-AlF-NOTA-MAL was simple. The reaction mixture of [^18^F]fluoride, aluminum chloride and NOTA-MAL was heated at 90-100°C for 10 min to obtain crude ^18^F-AlF-NOTA-MAL and then purified by semi-preparative HPLC. Also, the synthesis of ^18^F-FBEM required be anhydrous for the fist step, however, that of ^18^F-AlF-NOTA-MAL did not require be anhydrous. Therefore, it is easy and convenient to synthesize ^18^F-AlF-NOTA-MAL.

The yield of [^18^F]FBEM was low, less than 10% from [^18^F]fluoride without decay corrected and the synthesis lasted for about 100 min. And then the yield of [^18^F]FBEM-Cys-Annexin V was less than 70% from [^18^F]FBEM and the time of the reaction and purification of ^18^F-FBEM-Cys-Annexin V was over 40 min. Thus, the total yield of ^18^F-FBEM-Cys-Annexin V was about 5% from [^18^F]fluoride without decay corrected and consumed over 2 h. In contrast, the total yield of ^18^F-AlF-NOTA-MAL-Cys-Annexin V was about 15% from [^18^F]fluoride without decay corrected. And the preparation time of ^18^F-AlF-NOTA-MAL-Cys-Annexin V was just about 65 min, which was less than that of ^18^F-FBEM-Cys-Annexin V. It is important to reduce synthesis time and improve radiochemical yield of ^18^F site-specific labeling Annexin V derivatives.

Compared to ^18^F-FBEM-Cys-Annexin V,^18^F-AlF-NOTA-MAL-Cys-Annexin V showed similar biological properties, with the exception much higher renal metabolism. The peak value of kidney uptake of ^18^F-FBEM-Cys-Annexin V was 11%ID/g at 13 min p.i., which was less than that of ^18^F-AlF-NOTA-MAL-Cys-Annexin V (67%ID/g) at about 35 min p.i. The increase of kidney uptake of ^18^F-AlF-NOTA-MAL-Cys-Annexin V may due to hydrophilicity of ^18^F-AlF-NOTA. Some groups also reported higher kidney uptake of ^18^F-AlF-NOTA labeling peptide than that of ^18^F-FBEM labeling the same peptide [30].

*In vitro* and *in vivo* studies also showed that the radiotracer is a promising apoptosis imaging agent.

## MATERIALS AND METHODS

### General

Cys-Annexin V was supplied by Jiangsu Target Pharma Laboratories Inc. (Changzhou, China). ^18^F fluoride was obtained from the cyclotron (HM67, Sumitomo heavy industries, Ltd) of Jiangsu Institute of Nuclear Medicine by proton irradiation of ^18^O-enriched water. All other commercially obtained chemicals were of analytical grade and used without further purification.

A Waters high-performance liquid chromato-graphy (HPLC) system with a Waters 2998 photodiode array detector (PDA) and a semi-preparative C18 HPLC column (250×10mm, 5um, Chrom-Matrix Bio-Tech) was used for ^18^F-AlF-NOTA-MAL purification. The flow rate is 2 mL/min, and the mobile phase changed from 95% solvent A (0.1% trifluoroacetic acid in water) and 5% solvent B (0.1% trifluoroacetic acid in acetonitrile) (0-2 min) to 35% solvent A and 65% solvent B at 32 min. The UV absorbance was monitored at 218 nm, and the UV spectrum was checked with the PDA detector.

Analyzed HPLC was performed on Waters Breeze system with a TSK-GEL column (swG2000SWXL, 300 × 7.8 mm 5 µm, Tosoh Bioscience Co., Ltd, Shanghai, China). The absorbance was measured on the UV detector at 278 nm. Radioanalysis of the labeled compound was conducted using a Cd (Te) detector. The flow rate was adjusted to 1.0 mL/min and the isocratic mobile phase was 0.05 mol/L phosphate buffer (pH =7.0).

A microPET system (Inveon, Siemens Co. German) and a fluorescence microscope (Olympas X51, Tokyo, Japan) were used. The animal experiments in this study were approved by the Animal Care and Ethnics Committee of Jiangsu Institute of Nuclear Medicine.

### Radiosynthesis of ^18^F-AlF-NOTA-MAL

A 2 mL centrifuge tube was charged with 3µL of a solution of aluminum chloride (2 nM) in 0.5 M NaOAc (pH=4). Cyclotron target water containing [^18^F]fluoride (up to 100µL containing up to 3700 MBq) was added, followed by 200µg NOTA-MAL mono TFA, mono hexafluorophosphate salt (Chematech, Dijon, France) in 40µL of 0.5 M sodium acetate buffer (pH=4) and 200µL CH_3_CN. The resulting solution was heated at 90~100°C for 10 min. The reaction mixture was then cooled and injected onto a semi-preparative HPLC column. The radioactivity peak eluting at ~12 min was collected. The total synthesis time for ^18^F-AlF-NOTA-MAL was about 25 min and 925±23MBq (*n*=4) radiochemically pure ^18^F-AlF-NOTA-MAL was obtained from 14.8±0.5GBq ^18^F-fluoride.

### Labeling of Cys-Annexin V with ^18^F-AlF-NOTA-MAL

**Scheme 1:**
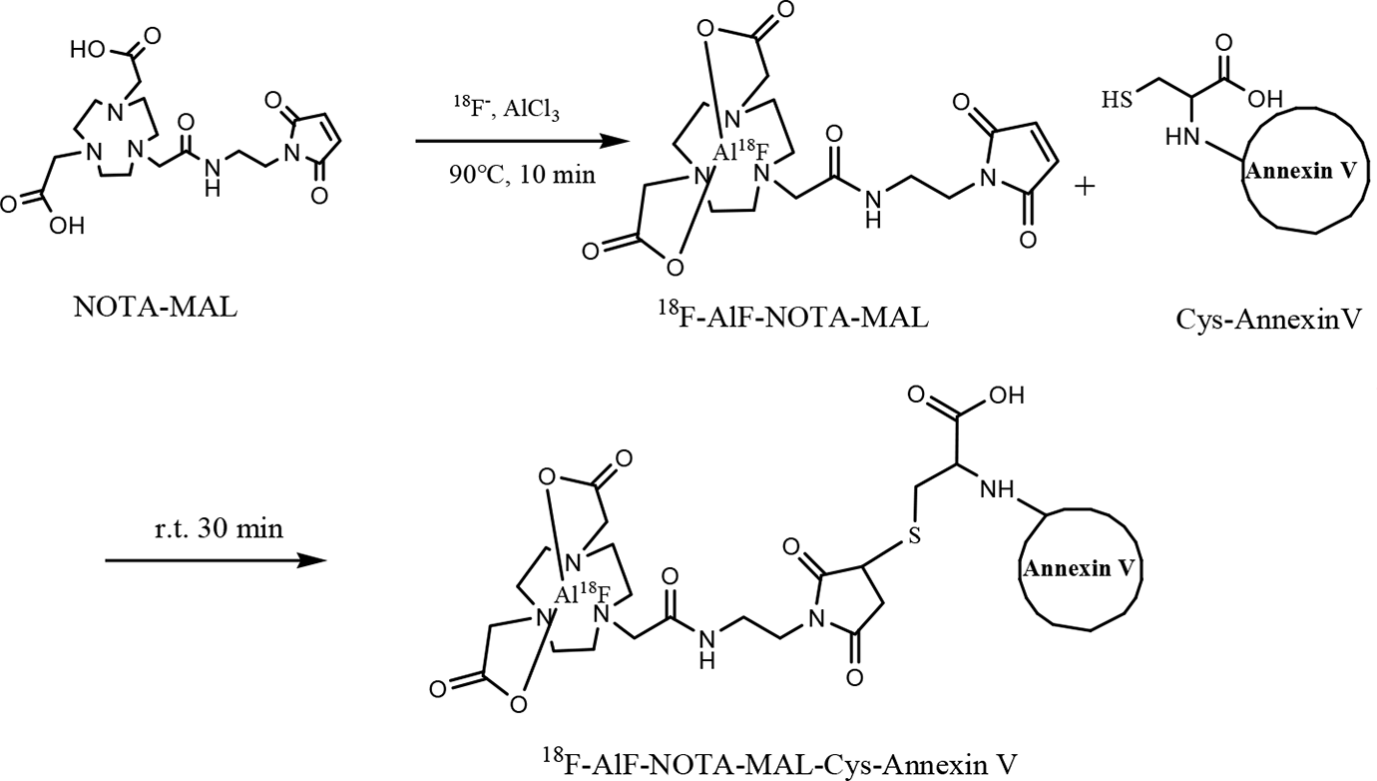
Synthesis of ^18^F-AlF-NOTA-MAL-Cys-Annexin V

The isolated ^18^F-AlF-NOTA-MAL (185–555 MBq) in 100µL was added to a solution of Cys-Annexin V (50~100 µg in 100µL, pH=7.2) PBS, and the mixture was allowed to react at room temperature for 15~30 min (Scheme 1) and loaded onto a NAP-5 column (GE Healthcare, Buckinghamshire, UK). The NAP-5 column was eluted with 250 µL portions of PBS. The most concentrated fraction containing the radiolabeled protein (fraction 3, 150~450MBq) was collected and used for the biological experiments.

### *In vitro* stability of ^18^F-AlF-NOTA-MAL-Cys-Annexin V

The *in vitro* stability of freshly prepared ^18^F-AlF-NOTA-MAL-Cys-Annexin V was investigated in PBS (0.1 mol/L, pH 7.2), human serum and cell culture media, respectively, for different time intervals (0,15, 30, 45, 60, 90, 120, 150, 180 min) at 37°C in a water bath.

### Bioactivity study

The bioactivity of ^18^F-AlF-NOTA-MAL-Cys-Annexin V was determined by its binding to erythrocytes, according to a previously reported procedure [32, 33]. In brief, ^18^F-AlF-NOTA-MAL-Cys-Annexin V at 10nmol/L final concentration was added to four tubes containing a final volume of 1 mL of buffer HNKGB (10mM HEPES-Na, pH 7.4, 136mM NaCl, 2.7 mM KCl, 5mM glucose, and 1mg/mL BSA) plus 2.5 mmol/L CaCl_2_. One tube then received 4.2×10^8^ erythrocytes with exposed phosphatidylserine in 100µL. The control tube then received an equal volume of buffer, and other two tubes then received 4.2×10^8^ erythrocytes with exposed phosphatidylserine and 50-fold of unlabeled Cys-Annexin V and 50-fold of BSA respectively in order to saturate and block any specific binding. Samples were incubated for 15 min at room temperature. After centrifugation at 8,320 *g* for 3 min, the radioactivity of the supernatant was measured with a packard-multi-prias gamma counter. The binding ratios were determined as follows: Radioactivity bound to erythrocytes (%)=(1-[radioactivity of supernatant in the presence of erythrocytes]/[radioactivity of supernatant in absence of erythrocytes]) ×100. All experiments were performed three times.

### Dynamic MicroPET imaging of mice

Four male ICR mice (25±2 g) were anesthetized with 1%–2% isoflurane, positioned prone, immobilized, and were injected via the tail vein with 0.2mL 3.7 MBq (100µCi) ^18^F-AlF-NOTA-Cys-Annexin V and imaged dynamically for 3h. The images were reconstructed using a two dimensional ordered-subset expectation maximization (2D OSEM) algorithm without correction for attenuation or scattering. For each scan, regions of interest (ROIs) were drawn over the liver and major organs using vendor software (ASI Pro 5.2.4.0) on decay-corrected whole-body coronal images. The radioactivity concentrations (accumulation) within the liver, heart and kidneys were obtained from mean pixel values within the multiple ROI volume and then converted to megabecquerel per milliliter per minute using the calibration factor determined for the Inveon PET system. These values were then divided by the administered activity to obtain (assuming a tissue density of 1 g/ml) an image-ROI-derived percent injected dose per gram (%ID/g).

### MicroPET images of rat model of apoptosis

Twelve male SD rats (258 ± 3g) were treated IV with 10 mg/kg cycloheximide to induce liver apoptosis and then were divided into three groups as treated group, blocking group and BSA-group. Other four male SD rats (259 ± 2g ) were treated IV with saline as the control group. 3 h after the treatment, rats of treated and control group were anesthetized with 1%–2% isoflurane and were injected via the tail vein with 0.2 mL (7.4 MBq, 200 µCi) ^18^F-AlF-NOTA-MAL-Cys-Annexin V. Rats of blocking and BSA group were coinjected with 7.4 MBq ^18^F-AlF-NOTA-MAL-Cys-Annexin V and blocking dose (5mg/kg body weight) of unlabeled Cys-Annexin V or bovine serum albumin (BSA), respectively. Ten-minute static scans were acquired at 1h after injection with a MicroPET (Inveon, Siemens), respectively. Immediately after MicroPET imaging, the livers were dissected. Then, using the livers, formalin-fixed paraffin-embedded specimens were prepared for Terminal deoxynucleotidyl transferase-mediated nick end labeling (TUNEL) staining.

### TUNEL staining

Because our imaging studies were designed to determine the uptake and biodistribution of ^18^F-AlF-NOTA-MAL-Cys-Annexin V after chemically induced apoptosis, it was important to confirm apoptosis in the livers of treated rats by independent methods that provide quantitative results. A marker of apoptosis was scored by performing a TUNEL assay that measures DNA fragmentation, a characteristic feature of apoptosis. Terminal deoxynucleotide transferase adds labeled nucleotides to the 3′ termini at double-stranded breaks in the fragmented DNA. TUNEL assays were performed according to the manufacturer's instructions, using the fluorescein-conjugated colorimetric TUNEL apoptosis assay kit (Beyotime Institute of Biotechnology, Shanghai, China). Briefly, slices were freed of paraffin through xylene and graded EtOH washes and then incubated with proteinase K (Beyotime Institute of Biotechnology) (2 mg/mL in 10 mmol/L Tris, pH 8.0). After proteinase digestion, the slides were equilibrated in pH 7.4 buffer, the terminal deoxynucleotide transferase enzyme and Biotin-dUTP labeling mix (Beyotime Institute of Biotechnology) were added, and the slides were incubated at 37 °C for 1 h in a humid chamber. The number of TUNEL-positive cells was counted on 10 randomly selected ×100 fields for each section by use of a Olympus fluorescence microscope.

### Statistical analysis

Quantitative data are expressed as mean ± SD. Means were compared using one-way analysis of variance (ANOVA) and Student's *t* test. P values <0.05 were considered statistically significant.

## CONCLUSIONS

Cys-Annexin V was successfully labeled with ^18^F via conjugated with ^18^F-AlF-NOTA-Mal, which is relative ease to radiochemical synthesis compared to ^18^F-FBEM. ^18^F-AlF-NOTA-MAL-Cys-Annexin V showed promising characterizations *in vitro* and *in vivo* for apoptosis imaging.
